# A candidate gene marker at the red kidney color locus (*Rk*) enables the development of slow-darkening pink beans

**DOI:** 10.1007/s00122-026-05256-z

**Published:** 2026-05-20

**Authors:** Caroline Osborne, Phillip E. McClean, Alvaro Soler Garzón, Atena Oladzad-Nejad, Rian Lee, Phillip N. Miklas, Juan M. Osorno, Justin D. Faris, Jayanta Roy

**Affiliations:** 1 Department of Plant Sciences, North Dakota State University, Fargo, ND USA; 2 Genomics, Phenomics, and Bioinformatics Program, North Dakota State University, Fargo, ND USA; 3 Irrigated Agriculture Research and Extension Center, Washington State University, Prosser, WA USA; 4 Crop Innovation Center in Cargill, Fort Collins, CO USA; 5 USDA-ARS Grain Legume Genetics and Physiology Research Unit, Prosser, WA USA; 6 USDA-ARS Cereal Crops Improvement Research Unit, Edward T. Schafer Agricultural Research Center, Fargo, ND USA

## Abstract

**Key message:**

** Identification of** ***Rk*** **candidate gene and diagnostic markers enabled precise introduction of the slow‑darkening (SD) allele into pink beans, producing stable SD phenotypes and expanding breeding utility across market classes.**

**Abstract:**

Common bean (*Phaseolus vulgaris* L.) is a globally important and nutritious crop with diverse market classes. Multiple genes control the seed coat color and patterns that characterize these market classes. Therefore, understanding the genetic control of seed coat color is critical for breeding purposes, especially when making crosses among different market classes. One such gene is *Rk*, the “red kidney” color gene. The recessive *rk*^*p*^ allele controls the characteristic color expression of the pink market class. Through GWAS analysis, sequencing candidate genes across multiple market classes, genetic mapping, and phylogenetic analysis, we determined the gene model PvUI111.02G213800, which encodes anthocyanidin reductase, is the most likely candidate for the *Rk* gene. Pink beans, along with several other market classes, suffer from postharvest seed coat darkening, a quality issue that causes significant economic losses and reduces consumer appeal and nutritional value. The recessive *P*^*sd*^ allele present in some pinto genotypes imparts a “slow-darkening” (SD) phenotype. To introduce the *P*^*sd*^ allele into the pink market class, crosses were made between three pink beans and the SD pinto cultivar ND Palomino. Among 2240 F_2_ plants screened using PACE markers targeting multiple genes (*P*^*sd*^, *rk*^*p*^, and *pi*) controlling pink color and slow-darkening, 32 individuals exhibited the desired alleles and SD pink genotype. The SD phenotype was verified via ultra-violet (UV) testing (*λ* = 254 nm), and field trials of F_5_ generation lines demonstrated their phenotypic stability. Parallel efforts are underway to incorporate the SD trait into light red kidney and cranberry beans, broadening its utility across market classes.

**Supplementary Information:**

The online version contains supplementary material available at 10.1007/s00122-026-05256-z.

## Introduction

Common bean (*Phaseolus vulgaris* L.) is an essential leguminous crop due to its high nutritional components such as protein, fiber, fat, minerals, and others. The protein content of beans ranges from 20 to 30%, making them comparable to meat (Brick et al. 2022). Considerable variability exists among common bean seed characteristics such as type, size, shape, seed coat color, and pattern, which define distinct market classes (Beninger and Hosfield 2003). Seed coat color, in particular, is a major contributor to the nutritional value of the crop. These colors are products of different classes and concentrations of flavonoids synthesized in the flavonoid pathway (Rodríguez Madrera et al. 2020). Flavonoids are associated with many health benefits, enhancing nutritional value and exerting positive effects on human health by functioning as antioxidants and exhibiting anti-diabetic, anti-obesity, and anti-mutagenic properties (de Mejía et al. 1999; Beninger and Hosfield 2003; Rodríguez Madrera et al. 2020).

Seed coat colors and patterns are major factors differentiating the varied dry bean market classes with regional consumer preference throughout the world. In several cases, multiple market classes can be preferred across different regions within a country, showing the strong local cultural influence (Singh 1999). Understanding the genetics of seed coat color is, thus, imperative for the improvement of different market classes, especially when trying to introgress genes of interest across different marker classes. Several genes that control seed coat color or pattern have already been cloned, including *P* (McClean et al. 2018) and *V* (McClean et al. 2022). In addition, candidate genes have been identified for the partial seed coat color genes *Bip*, *T,* and *Z* (Parker et al. 2024; McClean et al. 2024). There are three independent gene loci of particular importance in this study: *P*, *Rk*, and *Pi*. The *P* (pigment) gene on chromosome Pv07, often referred to as the basic color gene or the ground factor, determines if the seeds and flowers can produce pigment or not. When a plant is homozygous recessive for *p*, it will have white seeds and white flowers, which in almost all cases, trumps the action of any other color gene. The dominant allele *P* allows for the production of color (Bassett 2003). The “red kidney” (*Rk*) gene on Pv02, controls red seed coat color in a recessive manner (Bassett and Miklas 2003). The dominant form *Rk* gives no red color, whereas recessive alleles *rk*, *rk*^*p*^, *rk*^*d*^, and *rk*^*cd*^ confer various hues of red. The *rk* allele conditions the pink/testaceous color found in light red kidneys (LRK), the *rk*^*p*^ allele gives the pale pink color found in pink beans, and the *rk*^*d*^ allele gives the garnet brown color found in dark red kidneys (DRK). The *rk*^*cd*^ allele, as described by Bassett and Miklas (2003), gives a “convertible dark” phenotype which results in either pink or garnet brown seeds depending on the genotype of the *C* locus. Lastly, the *Pi* gene on Pv08, located in the complex *C* locus, controls expression of the pinto pattern. Genotypes with a dominant *Pi* allele express the pinto pattern, while those homozygous recessive for *pi* are without pinto pattern (Prakken 1977). Different types of seed coat patterns: marbling, striping, red color, light mottling, etc., are controlled by either alleles of a single locus “C” or tightly linked genes within the complex C locus because recombination between the patterns have not been validated (Bassett 2007). Until a multiple allelic series or tightly linked series of pattern genes is established, *Pi* (Prakken 1977) can be considered a temporary symbol for the gene in complex C locus, conditioning pinto pattern.

Seed coat color and pattern are important traits for consumers, but seed quality is also an important factor. Seed coats darken after harvest, a trait that is undesirable for both the industry and consumers (Mbiu et al. 2025). Darkened seeds are associated with longer cooking times and have been shown to have less bioavailable iron (Wiesinger et al. 2021). Postharvest seed coat darkening results in significant economic losses for farmers, as they must sell darkened seeds at a discounted price (Miklas et al. 2020). The severity of this problem varies between market classes and is most prevalent for market classes with light-colored seed coats such as pinto, pink, LRK, carioca, and cranberry, among others. Furthermore, the darkening effect is accentuated when seeds are exposed to high temperatures, humidity, and light (Junk-Knievel et al. 2008).

Pinto beans, the most widely produced market class in the USA (USDA-NASS 2024), have some of the most adverse postharvest darkening effects. However, this problem was addressed recently with the release of “slow-darkening” pinto bean cultivars, or SD pintos (Osorno et al. 2018, 2024). For example, in North Dakota, the largest producer of pinto beans in the US, industry estimates show that ~ 40% of the pinto cultivars grown are SD cultivars. Slow-darkening was initially thought to be controlled by a separate gene referred to as *Sd* (Elsadr et al. 2011). It has since been found to be a recessive allele at the *P* locus*,* denoted as *P*^*sd*^, which results in reduced production of proanthocyanidins and reduced seed coat darkening (Junk-Knievel et al. 2008; Islam et al. 2020). The SD trait was first identified at CIAT in various germplasm accessions. These lines were used in crosses to develop the first SD cultivar, “Pinto Saltillo” (Sanchez-Valdez et al. 2004; Singh 2006). Slow-darkening is now present in multiple pinto bean cultivars and widely adopted by the industry (Miklas et al. 2020). SD beans are particularly important in the Midwest, where the high humidity in the region accelerates darkening, compared to the more arid dry bean production regions further west. Several slow-darkening carioca cultivars have also been developed in Brazil (Carneiro et al. 2012). Carioca is the largest dry bean market class in Brazil. Carioca seeds are smaller than pinto but have a similar darker brown pattern on a lighter cream background.

Currently, only pinto and carioca beans have slow-darkening cultivars available, despite the fact that several other market classes such as pinks, LRKs, and cranberries suffer losses from seed coat darkening. Attempts have been made by several breeders to introduce the SD trait into these other market classes with no success, mostly due to the complex interactions among the genes controlling seed coat colors. This study aims to overcome this limitation by utilizing marker-assisted selection to ensure inheritance of both slow-darkening and desired seed coat color and pattern. The Andean and Middle American gene pools of *P. vulgaris* have partial reproductive isolation which dictates introgressing traits within gene pools when possible (Papa et al. 2006). The pinto and pink market classes are both from race Durango/Jalisco in the Middle American gene pool (Kelly 2010), and thus our primary objective was to transfer the SD trait from pinto into pink beans.

To develop SD pink beans, molecular markers were required to select three key seed traits: reduced postharvest darkening, pink seed coat color, and no pinto pattern. Progeny homozygous for the *P*^*sd*^, *rk*^*p*^, and *pi* alleles were expected to exhibit slow-darkening, solid pale pink seeds, and no pinto pattern, respectively. While pre-existing PCR Allele Competitive Extension (PACE) markers linked to *P*^*sd*^ (Alvares et al. 2019) and *Pi* (Roy et al. 2025a) were available, no diagnostic marker exists for selecting the solid pink *rk*^*p*^ allele. Thus, identification of the *Rk* gene and development of an allele-specific marker for *rk*^*p*^ were essential steps toward enabling marker-assisted selection (MAS) for SD pink beans. To address this need, the current study was designed to meet the following objectives: (i) define the genomic region underlying the *Rk* locus by GWAS and identify its candidate gene; (ii) validate the candidate gene through gene expression analysis, linkage mapping, phylogenetic analysis, and sequence comparison; (iii) develop diagnostic PACE markers for the *Rk* locus; (iv) implement MAS using *P*^*sd*^, *pi*, and *rk*^*p*^ markers to develop SD pink beans; and (v) establish a groundwork for applying these and other seed coat color and pattern gene markers for extending the slow-darkening trait to Andean market classes such as LRK and cranberry.

## Materials and methods

### Plant materials and phenotyping

The Durango Diversity Panel (DDP; *n* = 192), a subset of the Middle American Diversity Panel (MDP; *n* = 280) (Moghaddam et al. 2016), was used as plant materials for the GWAS analysis. The DDP panel includes pinto, pink, Durango red, great northern, and navy bean market classes. Only the genotypes belong to the pinto (*n* = 92), pink (*n* = 22), and Durango red (*n* = 22) market classes were used in the GWAS analysis. Phenotyping for the GWAS was based on the known genotypic differences among these three market class groups. The panel was scored in a binomial manner: pinto beans (*n* = 92; genotype: *G B v Rk Pi*) vs a pooled group consisting of solid-colored pink beans (*n* = 22; genotype: *G b v rk*^*p*^* pi*) and Durango red beans (*n* = 22; genotype: *G b v* *rk*^*cd*^ *pi*). These two pools differ specifically at the *B*, *Rk*, and *Pi* loci. The phenotype scores used for the GWAS are provided in Supplementary Table S1.

### Genotyping and variant calling

Fresh young leaves at trifoliate stage were collected from a single plant per genotype, and genomic DNA was extracted using the Mag‑Bind Plant DNA Plus Kit (Omega Bio‑Tek, Norcross, Georgia, USA). The DDP panel (*n* = 192) was resequenced in 150‑bp paired‑end mode at approximately 8× depth by the HudsonAlpha Institute for Biotechnology (Huntsville, AL). Sequencing reads were aligned to the UI111 v1.1 reference genome (https://phytozome-next.jgi.doe.gov/info/PvulgarisUI111_v1_1) using BWA‑MEM (Li 2013) with default parameters. The resulting BAM files were sorted with SAMtools v1.15.1 (Li et al. 2009), and duplicate reads were removed using Picard (http://broadinstitute.github.io/picard). Variant calling was performed using GATK v3.3 (McKenna et al. 2010). Multiallelic SNPs and SNPs with a read depth ≤ 5 were removed using VCFtools (Danecek et al. 2011). SNPs with ≤ 20% missing data were imputed using the likelihood‑based method implemented in fastPHASE v1.3 (Scheet and Stephens 2006), resulting in 2,051,922 SNPs. Finally, SNPs with a minor allele frequency (MAF) < 0.05 were excluded prior to GWAS analysis. Some of the resequenced DDP genotypes were used in this study for the creation of *Rk* markers (Table 1).

**Table 1:** Genotypes used to create diagnostic PACE markers for *Rk*

Market class	Genotypes	Sequence origin^a^
Durango red	NW63, Common Red, UI37, USRM20	DDP resequencing
Pink	Gloria, CDC Rosalee, Pink Floyd, Yolano, USWA61, Viva	DDP resequencing
DRK	Montcalm, MDRK, RedHawk, Royal Red	Amplicon sequencing
LRK	Sacramento, USLK1, CELRK, Pink Panther	Amplicon sequencing
Red mottled	Rozi Koko, Maalasa, PI310511 (Calima)	Amplicon sequencing
Small red	Kibumbula, G1368, G20729, G23070	Amplicon sequencing
Cranberry	USCR-CBB-20, Capri, Bellagio	Amplicon sequencing
Black	Labor Ovalle, 5-593	Phytozome
Pinto	UI111	Phytozome
Liborino	G19833	Phytozome

^a^Source of the *Rk* DNA sequence. Sequences were captured from resequencing data of the DDP mapped to the UI111 reference genome (DDP resequencing; https://phytozome-next.jgi.doe.gov/info/PvulgarisUI111_v1_1), amplicon sequencing data collected during this project (amplicon sequencing), or the annotation of the four common bean reference genomes (Phytozome; https://phytozome-next.jgi.doe.gov/)

### Genome-wide association study (GWAS)

A binary GWAS was performed using the phenotype classification described above to identify genomic regions associated with the *Rk*, *B*, and *Pi* loci. A total of 1,170,272 SNP loci with MAF > 0.05 from 136 genotypes were included in the GWAS. A principal component analysis (PCA) using the R *prcomp* function implemented in the GAPIT R package (Lipka et al. 2012) was used to estimate population structure. The centered relatedness algorithm to determine population relatedness was computed in GEMMA (Zhou and Stephens 2012). A general linear mixed model was performed in GEMMA that included population structure and relatedness as fixed and random effects, respectively. To determine if the SNP effect size was significantly different from zero, a Wald test was used (Wald 1943). SNPs within the lower 0.01% tail of the SNP *P* value were considered significant. A Manhattan plot was created using SNPs with a minor allele frequency (MAF) > 0.05 with the *mhtplot* function in the R package gap (Zhao 2007).

To further investigate potential epistatic interactions between the *Rk* and *C* loci, a targeted GWAS was conducted using two genetically defined groups: i) solid-colored pink beans (*n* = 22) and (ii) Durango red beans (*n* = 22). A total of 1,010,744 SNPs were used for the analysis, and GWAS was performed using the same filtering criteria, statistical models, and population structure controls described above.

### Candidate gene selection

The ± 50 kb region surrounding the *Rk* peak on Pv02 was searched for genes associated with flavonoid production. Two tandemly duplicated genes, PvUI111.02G213700 and PvUI111.02G213800, were selected as prospective candidates. Both genes were annotated as anthocyanidin reductase (ANR).

RNA-seq data was analyzed from the black-seeded 5-593 genotype seed coat tissue collected at four stages of seed coat development (Roy et al. 2025b). The line 5-593 (genotype: *T Z Bip P* [*C r*] *J G B V Rk Gy sal*), developed by Dr. Mark J. Bassett at the University of Florida, carries dominant alleles controlling seed coat color and pattern for all but two of the listed genes (Bassett 1998). The expression data were compared and due to the lack of expression of the 5-593 homolog of PvUI111.02G213700, PvUI111.02G213800 was selected as the candidate gene for *Rk*.

### Phylogenetic analysis of *Rk* gene alignment of orthologs

A neighbor-joining (NJ) phylogenetic tree was constructed using the *Rk* protein (PvUI111.02G213800) together with its orthologous proteins. Orthologs were identified through blastp searches against Phytozome 13 (https://phytozome-next.jgi.doe.gov/) and NCBI databases. A total of 61 amino acid sequences annotated as ANR proteins, representing 57 species previously used for *V* gene analysis by McClean et al. (2022), were included in the analysis (Supplementary Table S2). Protein sequences were aligned using the MUSCLE algorithm (Edgar 2004) as implemented in MEGA11 (Tamura et al. 2021). The Jones–Taylor–Thornton substitution model was applied for tree development. The NJ tree was generated with 1000 bootstrap replicates to assess branch support.

### Genetic linkage mapping

A bi-parental F_5_-derived population of recombinant inbred line (RIL) was developed from a cross between G122 (cranberry) and Montcalm (dark red kidney), hereafter referred to as the GM population, (Bello et al. 2014) was used for mapping of the *Rk* locus. G122 has the genotype *T P* [*C*^*st*^* R pi*] *J g b v*^*lae*^* Rk,* while Montcalm has the genotype *T P* [*c*^*u*^* r pi*] *J g B v rk*^*d*^. This population consists of 108 RILs and was used to assess recombination rates in the genomic region surrounding the *Rk* candidate gene. The GM RILs were visually scored based on seed coat color. Lines exhibiting the DRK market class characteristic of garnet brown seed color were classified as *rk*^*d*^, while all other seed coat types were classified as *Rk ***(**Supplementary Fig. S1**)**. Detailed phenotypic information about each GM RIL can be found in Supplementary Table S3.

Whole-genome resequencing data were aligned to the UI111 *P. vulgaris* reference genome to create bam file for visualization. Integrative Genomics Viewer (IGV) (Robinson et al. 2011) was used to identify polymorphisms between the GM RIL parental lines, G122 and Montcalm. PACE markers were designed approximately every 200 kb across the genomic interval identified in the GWAS analysis, including flanking regions on both the left and right sides of the *Rk* locus. A total of 18 PACE markers polymorphic between G122 and Montcalm were designed for the region spanning 37,339,802 bp to 41,080,767 bp (UI111) on chromosome Pv02 for fine-mapping (Supplementary Table S4). These PACE markers were subsequently used to genotype and map the *Rk* gene in the GM population (Supplementary Table S3).

The SNP allelic discrimination genotyping data of the GM RIL population were analyzed using MapDisto v.2.1.8.7 (Heffelfinger et al. 2017). Linkage group was created using the Kosambi mapping function with a minimum LOD (logarithm of odds) threshold of 5.0 and a maximum classical recombination fraction of 0.3. Marker ordering was performed using the “Seriation II” algorithm under the “order a linkage group” command. The order was refined and optimized further with the “check inversions” and “ripple order” commands.

### DNA isolation

Trifoliate young leaf tissue or embryo tissue collected from each member of the GM RIL population (Supplementary Table S3) and amplicon sequencing genotypes were used for DNA extraction (Table 1). DNA was extracted using the Mag-Bind DNA Plus kit (Omega Bio-Tek, Norcross, Georgia, USA). The same strategy was employed for leaf tissue harvested from F_2_ progeny resulting from crossing ND Palomino with the other genotypes. DNA extractions on leaf tissue were performed using half-reaction mix.

### DNA amplicon sequencing of the *Rk* candidate gene

The structure of the *Rk* gene model PvUI111.002G213800 from UI111 reference genome was used to develop primers for PCR amplifications (Supplementary Table S5). DNA fragments were amplified in a 20 µL volume reactions with an amplification protocol with 45 cycles and annealing temperatures specific to each primer pair. PCR products were run on a 1.5% agarose gel, and the DNA fragments were extracted using the NEB Monarch Gel Extraction kit (https://www.neb.com/products/t1020-monarch-dna-gel81 extraction-kit). The purified DNA fragments were Sanger sequenced by Eton Bioscience Inc. (https://www.etonbio.com/). The coding region was assembled from the sequence fragments using the Staden Package (Staden 1996).

### Diagnostic marker development and PCR allele competitive extension (PACE) analysis

To develop diagnostic PACE markers differentiating *Rk* alleles, whole-genome shot gun sequencing data along with amplicon sequencing data from genotypes across multiple market classes were analyzed (Table 1). Three missense SNPs within the *Rk* candidate gene model were identified as being associated with specific market classes. These SNPs were used to design three allele-specific PACE markers (Supplementary Table S6). These PACE markers were applied to determine *Rk* genotypes for the crosses created in this study and in two diversity panels: the Middle American Diversity Panel (MDP; *n* = 284; Supplementary Fig. S2; Supplementary Table S7) and the Andean Diversity Panel (ADP, *n* = 256; Supplementary Table S8). Seed type classifications for both panels were obtained from McClean et al. 2024.

PACE assays were performed in a total of 8 μL reaction volume, each containing 2 μl (approximately 10 ng/μl) of genomic DNA, 0.15 μl of primer mix (12 μM of each allele-specific forward primer and 30 μM of the common reverse primer), 4 μl of 3CR Bioscience PACE Master Mix Standard Rox, and 1.85 μl of H_2_O. PCR amplifications followed the protocol described in McClean et al. (2024). Following post-PCR, end-point fluorescence was measured using the CFX Opus 96 Real-Time PCR System (Bio-Rad®, Inc., Hercules, CA, USA). The relative fluorescent unit (RFU) intensities data were then analyzed for allelic discrimination using the CFX Maestro v2.3 software (Bio-Rad®).

### Development of populations to introduce the *P*^*sd*^ allele into pink beans

The SD pinto cultivar ND Palomino was jointly developed by North Dakota State University (NDSU) and the USDA-ARS and released in 2016 (Osorno et al. 2018). ND Palomino (*P*^*sd*^* Rk Pi*) was used as the SD donor male parent and crossed to three pink-seeded parents, Rosetta (Kelly et al. 2012), PK16-1 (breeding line from the USDA-ARS bean breeding program at Prosser-WA), and ND112929, a breeding line from NDSU in the NDSU Jack Dalrymple greenhouse complex in Fargo, ND. All three regular-darkening pink parents possess the *P rk*^*p*^* pi* genotype. F_1_ seeds from these crosses were advanced to F_2_ generation.

The F_2_ populations from each cross were grown, and DNA was extracted from leaf tissue of individual F_2_ plants for genotyping using three PACE markers targeting the *Rk*,* P*^*sd*^, and *Pi* loci associated with seed coat color and pattern. These markers were used to identify plants homozygous for the desired alleles. The F_3_ seeds derived from selected F_2_ plants were evaluated for seed coat color and pattern through phenotypic scoring with UV light. Finally, 16 out of 28 viable F_3_ lines were advanced to the F_5_ generation.

### Ultra-violet (UV) screening for the slow-darkening trait

To evaluate postharvest seed coat darkening, seeds from F₂ (greenhouse-grown), and F₅ (field-grown) generations SD pink bean lines were subjected to UV light exposure to induce postharvest darkening. Seed samples (5–20 seeds) were arranged in a single layer and placed approximately 20 cm beneath a germicidal UV light source (*λ* = 254 nm) for 120 h. This protocol was adapted from Islam et al. (2020), with modifications including a shorter distance from the light source and extended exposure duration to enhance the visibility of darkening effects.

Following treatment, the UV-exposed surface of each seed was visually compared to the unexposed side and to untreated control seeds. Genotypes with known seed coat darkening behaviors were included as controls. This comparative approach enabled clear differentiation between slow-darkening and regular-darkening phenotypes and validated trait expression across generations.

## Results

### GWAS

**Fig. 1:**
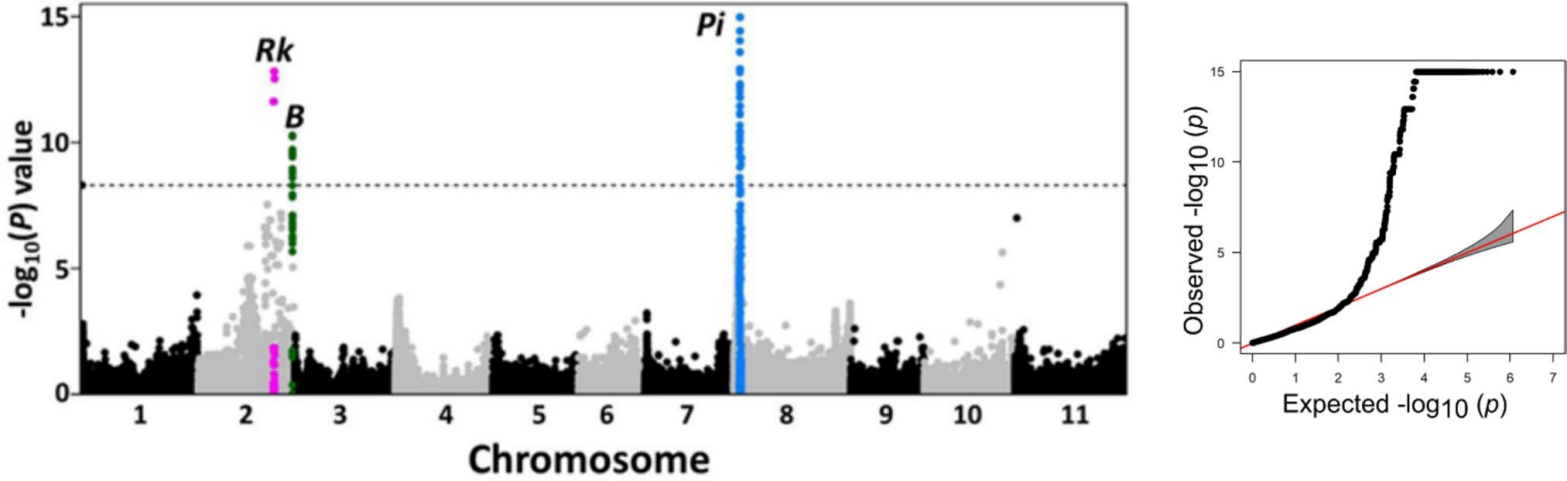
Manhattan plot and Q-Q plot for the GWAS of red seed coat color. The Manhattan plot shows a strong peak for *Rk* in chromosome 2. Associations were also found for *Pi* and *B*. The dotted line represents the 0.01% significance threshold

The GWAS conducted with 1,170,272 SNP loci and two seed coat categories for the DDP: pinto (*n* = 92) and a pooled group of solid-colored pink and Durango red beans (*n* = 44), identified candidate genomic regions for the *Rk*, *B*, and *Pi* loci that aligned fully with expectations. A strong association corresponding to the target gene *Rk* was detected toward the distal end of chromosome Pv02 (Fig. 1). A cluster of 22 SNPs spanning a 0.8 Mb interval (39.3–40.1 Mb) on Pv02 chromosome were identified and used for candidate gene exploration of the *Rk* gene (Supplementary Table S9). A second peak was also observed at the distal end of Pv02. Because the *B* locus has historically been linked to the *I* virus resistance gene (Temple and Morales 1986), which also maps to chromosome Pv02. Preliminary data (not shown) suggest that this peak is positioned with the *B* locus. Apparently, pintos in the DDP are predominantly dominant *B* while reds and pinks are recessive* b*. The analysis also showed, as expected, a distinct peak for the seed coat pattern gene *Pi* at the proximal end of chromosome Pv08 (Roy et al. 2025a) where the complex *C* locus is located.

The targeted GWAS comparing solid-colored pink beans and Durango red beans revealed a prominent association peak on chromosome Pv08 (Supplementary Fig. S3). This genomic interval corresponds to the known physical location of the complex C locus, supporting the hypothesis of Bassett and Miklas (2003) that the darker background pigmentation of Durango red beans may result from an interaction between the *C* gene and the *rk*^*cd*^ allele at the *Rk* locus. An additional peak was detected on chromosome Pv07; however, examination of annotated genes within this region did not identify any genes associated with the flavonoid biosynthesis pathway or with known seed coat color loci.

### Candidate gene selection

The 0.8 Mb region surrounding the *Rk* peak genomic interval was searched for genes associated with flavonoid production. Two tandemly duplicated gene models, PvUI111.02G213700 and PvUI111.02G213800, were selected as prospective candidates. The ortholog for these genes in the model organism Arabidopsis is *AT1G61720*, deemed the *BANYULS* (*BAN*) gene. This gene has the same negative regulatory effect on flavonoid synthesis as the *Rk* gene (Albert et al. 1997). The *BAN* gene encodes for anthocyanidin reductase (ANR) (Xie et al. 2003).

RNA-seq data of seed coat tissue from the 5-593 genotype sampled across four development stages of seed coat color formation showed that Pv5-593.02G212800, the homolog for PvUI111.02G213800, was expressed at all stages (Table 2**)** (Roy et al. 2025b). In contrast, the 5-593 homolog of PvUI111.02G213700, Pv5-593.02G212700, had virtually no expression across all stages. Based on these expression profiles, PvUI111.02G213800 was chosen as the most plausible *Rk* candidate gene.

**Table 2:** RNA-seq based expression profiles of *Rk* candidate genes in seed coat tissue of genotype 5-593 across four developmental stages [adapted from Roy et al. (2025b)]

Gene model in 5-593	Gene model in UI111	Stage 1^a^	Stage 2^a^	Stage 3^a^	Stage 4^a^
Pv5-593.02G212700	PvUI111.02G213700	0.03	0	0.01	0.03
Pv5-593.02G212800	PvUI111.02G213800	738.9	542.47	217.24	123.57

^a^Values represent the mean of three biological replicates measured in transcripts per million (TPM). Stage 1 corresponds to the earliest stage of seed coat color development, while stage 4 represents a nearly mature seed coat. Expression data were generated from genotype 5-593; homologous gene models from UI111 are provided for reference

### Phylogenetic analyses of *Rk* gene and their orthologs

**Fig. 2:**
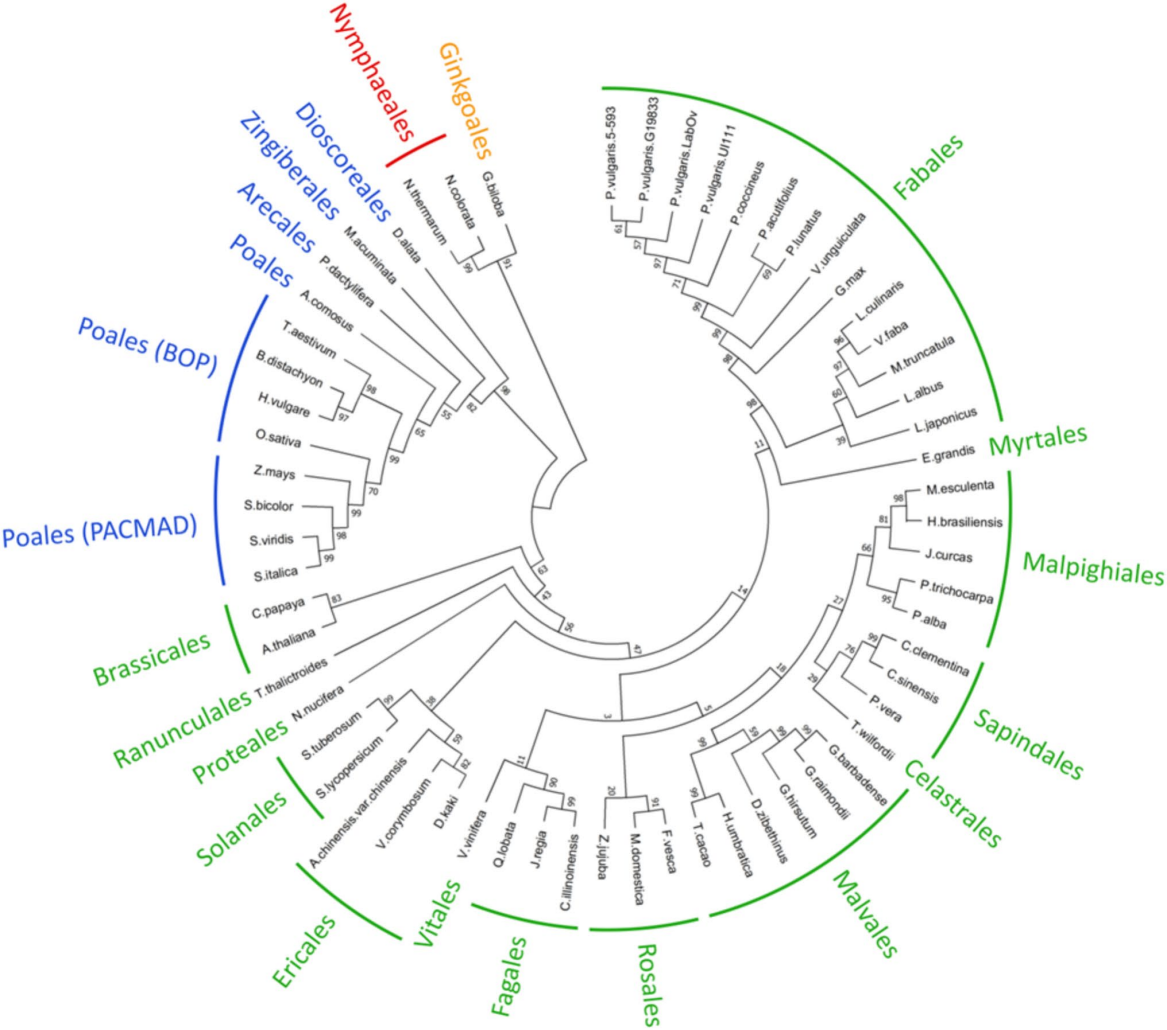
A neighbor‑joining (NJ) phylogenetic tree of anthocyanidin reductase (ANR) orthologs from 57 plant species spanning the diversity of land plants. The *Rk* candidate protein (P.vulgaris.UI111: PvUI111.02G213800) clusters within the Fabales clade, reflecting high conservation among legumes. Colors denote major plant orders. Bootstrap values (1000 replicates) indicate branch support are shown

To evaluate the evolutionary conservation of the *Rk* candidate gene, a neighbor‑joining (NJ) phylogenetic tree was constructed using the *Rk* protein (PvUI111.02G213800) and 60 additional ANR orthologs representing 57 species. Orthologs were identified via blastp searches across the *Phaseolus* genus and broader angiosperm and gymnosperm lineages. The tree (Fig. 2) reveals strong clustering of *Phaseolus* species, with the Rk protein showing complete identity to its orthologs from the other three common bean reference genome annotations (G19833 v2.1, 5-593 v1.1, and Labor Ovalle v.1.1), and 99% identity to tepary bean (*P. acutifolius*), and 98% to scarlet runner bean (*P. coccineus*). *Rk* was 94% and 90% identical to cowpea (*Vigna unguiculata*) and soybean (*Glycine max*) orthologs, respectively.

In soybean, two *ANR* orthologs were identified in the Williams82 reference genome, Glyma.08G062000 (*ANR1*) and Glyma.08G062100 (*ANR2*). Glyma.08G062000 (*ANR1*) has been associated with red‑brown seed coat pigmentation in soybean (Kovinich et al. 2012). The *Rk* protein shared 90% identity with *ANR1* and 79% with *ANR2*. These identity values and clade placements in Fig. 2 reflect taxonomic distances and support the functional conservation of ANR‑type enzymes in seed coat color regulation.

### Genetic linkage mapping

To refine the genomic region surrounding the candidate gene, PvUI111.02G213800, genetic linkage mapping was performed using the GM RIL population. This population was chosen because the parents, G122 and Montcalm, have contrasting *Rk* alleles: *Rk* and *rk*^*d*^, respectively. The mapping interval spanned from 37,339,802 bp to 41,080,767 bp on Pv02, extending beyond the GWAS-defined peak interval (39.3–40.1 Mb) in both directions to ensure comprehensive coverages.

**Fig. 3:**
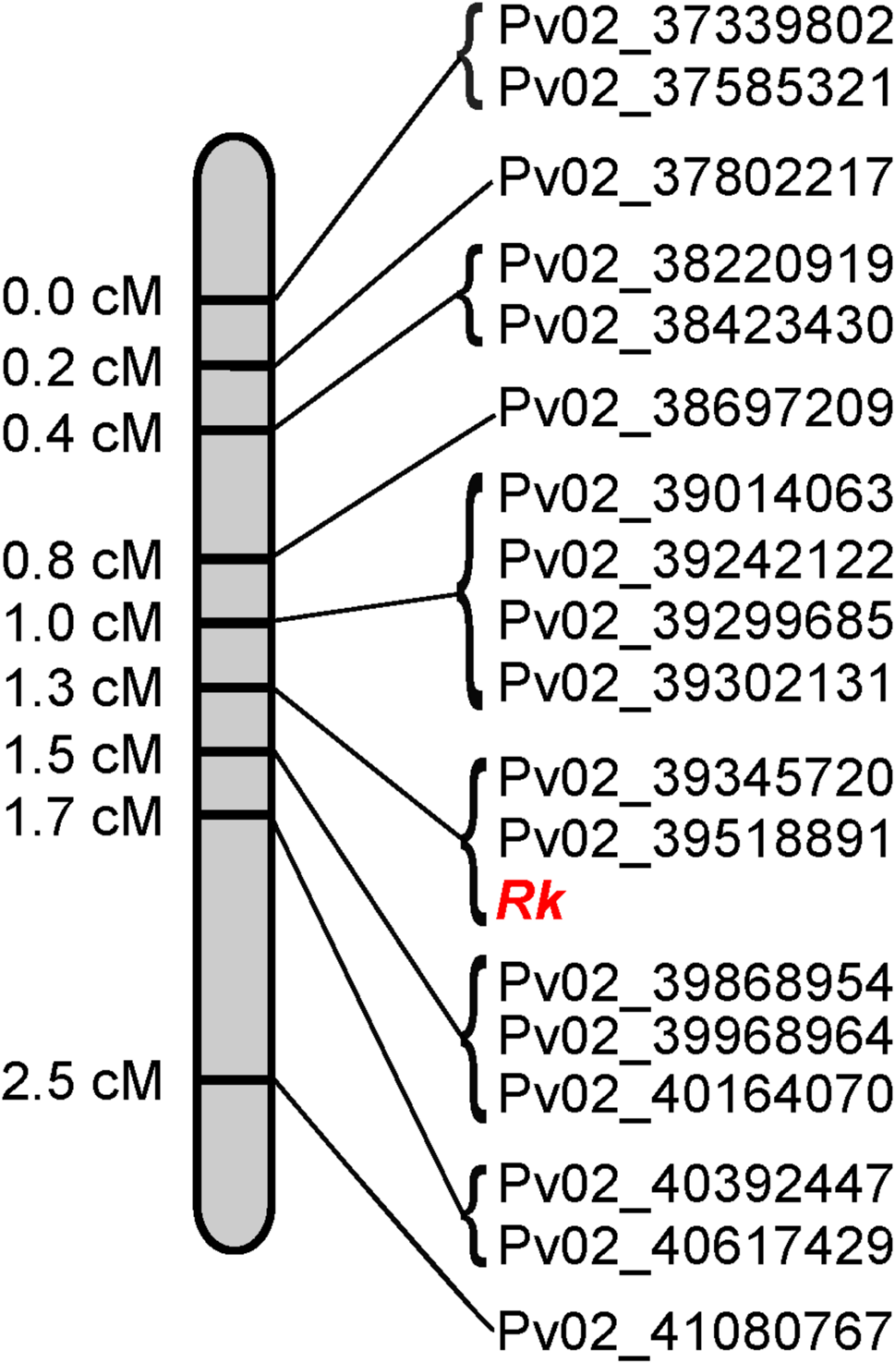
Genetic linkage mapping of the *Rk* locus in the G122 x Montcalm recombinant inbred line population. The physical position of SNP markers denoted after Pv02 correspond to the coordinates in the UI111 reference genome. The mapping software’s approximation of genetic distance is indicated in cM on the left. The *Rk* locus, highlighted in red, cosegregates with two markers: Pv02_39345720 and Pv02_39518891

Polymorphic SNPs between the two parents were identified approximately every 200 kb in either direction flanking the candidate gene and converted into PACE markers. This strategy yielded nine proximal markers and six distal markers relative to PvUI111.02G213800 gene model. Additionally, two flanking markers were placed roughly 500 kb beyond the nearest marker at each end of the interval. Finally, the PACE marker denoted Pv02_39518891, located inside the candidate gene, was designed. In total, 18 PACE markers were used to genotype all 108 GM RILs and both parental lines. Linkage analysis revealed that two markers cosegregated with the *Rk* gene, including Pv02_39518891, providing strong support for PvUI111.02G213800 as the candidate gene. The resulting genetic map is presented in Fig. 3.

### Diagnostic *Rk* PACE marker development

**Fig. 4:**
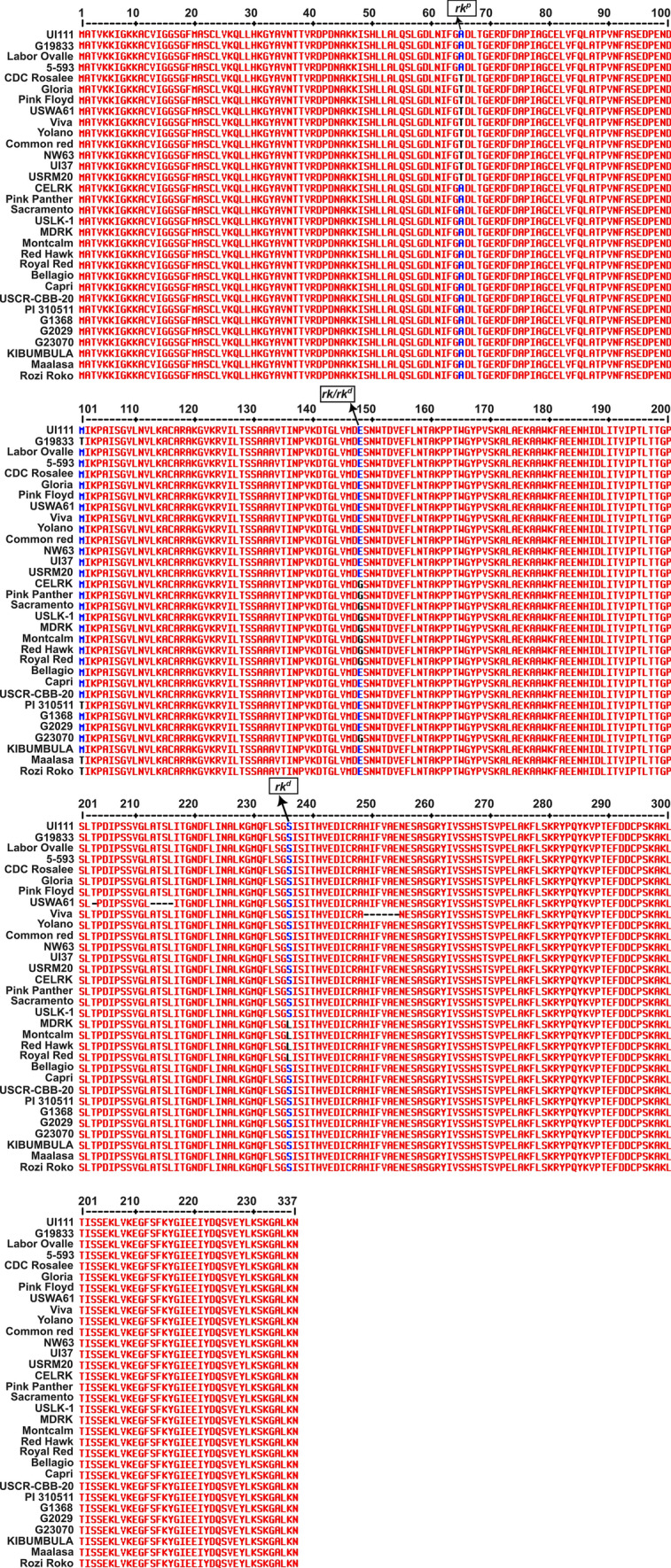
Amino acid alignment of proteins representing distinct genotypes of the *Rk* gene model PvUI111.02G213800. Three SNPs associated with different *Rk* alleles that result in amino acid substitutions are highlighted

**Fig. 5:**
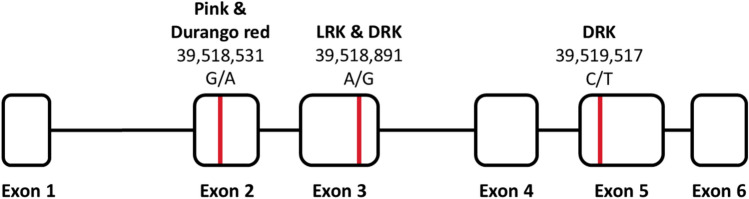
The *Rk* candidate gene PvUI111.02G213800 and associated diagnostic SNPs. PvUI111.02G213800 spans from 39,517,944 to 39,519,962 bp on chromosome Pv02. The three SNPs used to create relevant PACE markers are highlighted in red. SNP positions (relative to UI111) and the associated market class(es) are shown above each site. For each SNP, the first listed nucleotide represents the allele present in UI111, while the second represents the allele found in the associated market class(es)

The Coding DNA Sequences (CDS) for the *Rk* gene PvUI111.02G213800 were searched across multiple market classes (Table 1) to identify nucleotide polymorphisms. Three SNPs were found that resulted in amino acid substitutions distinguishing different *Rk* alleles correlated with specific market classes. All pink and Durango red genotypes shared identical CDS sequences and carried a SNP G193A at position 39,518,531 bp which led to an A65T amino acid substitution. Both LRKs and DRKs genotypes shared a SNP A443G at 39,518,891 bp, corresponding to E148G substitution. Additionally, a DRK-specific SNP C707T was identified at 39,519,517 bp, leading to a S236L substitution (Fig. 4). These diagnostic SNPs are summarized in Table 3, and visualized in Fig. 5, providing molecular markers for distinguishing *Rk* alleles across market classes.

**Table 3:** Missense SNPs distinguishing different market classes in the *Rk* candidate gene model PvUI111.02G213800

Market Class	Genotype	*rk*^*p a*^		*rk* and *rk*^*d a*^		*rk*^*d a*^	
		Pv02_39518531^b^		Pv02_39518891^b^		Pv02_39519517^b^	
		SNP	AA^c^	SNP	AA^c^	SNP	AA^c^
Pinto	UI111	G	A	A	E	C	S
Liborino	G19833	G	A	A	E	C	S
Black	Labor Ovalle	G	A	A	E	C	S
	5-593	G	A	A	E	C	S
Cranberry	USCR-CBB-20	G	A	A	E	C	S
	Capri	G	A	A	E	C	S
	Bellagio	G	A	A	E	C	S
Pink	Gloria	**A**	**T**	A	E	C	S
	CDC Rosalee	**A**	**T**	A	E	C	S
	Pink Floyd	**A**	**T**	A	E	C	S
	Yolano	**A**	**T**	A	E	C	S
	USWA61	**A**	**T**	A	E	C	S
	Viva	**A**	**T**	A	E	C	S
Durango red	NW63	**A**	**T**	A	E	C	S
	Common Red	**A**	**T**	A	E	C	S
	UI37	**A**	**T**	A	E	C	S
	USRM20	**A**	**T**	A	E	C	S
LRK	Sacramento	G	A	**G**	**G**	C	S
	USLK1	G	A	**G**	**G**	C	S
	CELRK	G	A	**G**	**G**	C	S
	Pink Panther	G	A	**G**	**G**	C	S
DRK	Montcalm	G	A	**G**	**G**	**T**	**L**
	MDRK	G	A	**G**	**G**	**T**	**L**
	RedHawk	G	A	**G**	**G**	**T**	**L**
	Royal Red	G	A	**G**	**G**	**T**	**L**

^a^*rk*^*p*^ allele is designates the pink and Durango red phenotype*; rk* designates the light red kidney; and *rk*^*d*^ designates the dark red kidney phenotype at the* Rk locus*

^b^SNP locations are based on the reference genome UI111 (https://phytozome-next.jgi.doe.gov/info/PvulgarisUI111_v1_1). Above each SNP is the allele(s) of *Rk* associated with it. The amino acid encoded by the codon that includes each SNP is also shown

^c^AA indicates amino acid. The highlighted nucleotide in the SNP column and the corresponding amino acid in the AA column represent the missense SNP and amino acid substitution associated with each allele-specific locus in the respective market classes

Three PACE markers targeting these SNPs were developed and validated using the diverse MDP and ADP panels. The PACE marker for the *rk*^*p*^ allele (*rk*^*p*^-PACE-PinkFloyd-SNP-39518531-UI111) *[Marker name components indicate*: allele targeted (*rk*^*p*^), assay type (PACE), source genotype (Pink Floyd), variant type (SNP), physical position (39,518,531 bp), and reference genome (UI111)] was 100% diagnostic in classifying pink and Durango red beans from other market classes in both panels. The marker targeting the shared SNP between LRK and DRK (*rk*-PACE-Celrk-SNP-39518891-UI111) genotypes successfully detected both market classes in the ADP panel, with few exceptions, and represented an alternate allelic state in the MDP panel. The DRK-specific PACE marker (*rk*^*d*^-PACE-Montcalm-SNP-39519517-UI111) successfully differentiated the DRK genotypes from LRK in the ADP, and from the other market classes in both panels (Table 3; Supplementary Tables S7 and S8).

In addition to these diagnostic SNPs, three other polymorphisms detected in this study could not be definitively associated with an *Rk* allele. Two SNPs at position 39,518,497 and 39,519,757 bp within the *Rk* gene were silent mutations, although they may still have associations with particular market classes. These SNPs were present in multiple bean types, including the small red landraces. Attempts to develop a marker for identifying small red landraces using these polymorphisms in combination with other markers proved unsuccessful. Furthermore, analysis of the red mottled market class revealed a missense SNP at position 39,518,750 bp, potentially associated with red mottled beans (Supplementary Table S10). A marker was developed for this SNP to identify red mottled beans, but it also failed to provide reliable classification.

### Introduction of the ***P***^***sd***^ allele into pink beans

**Fig. 6:**
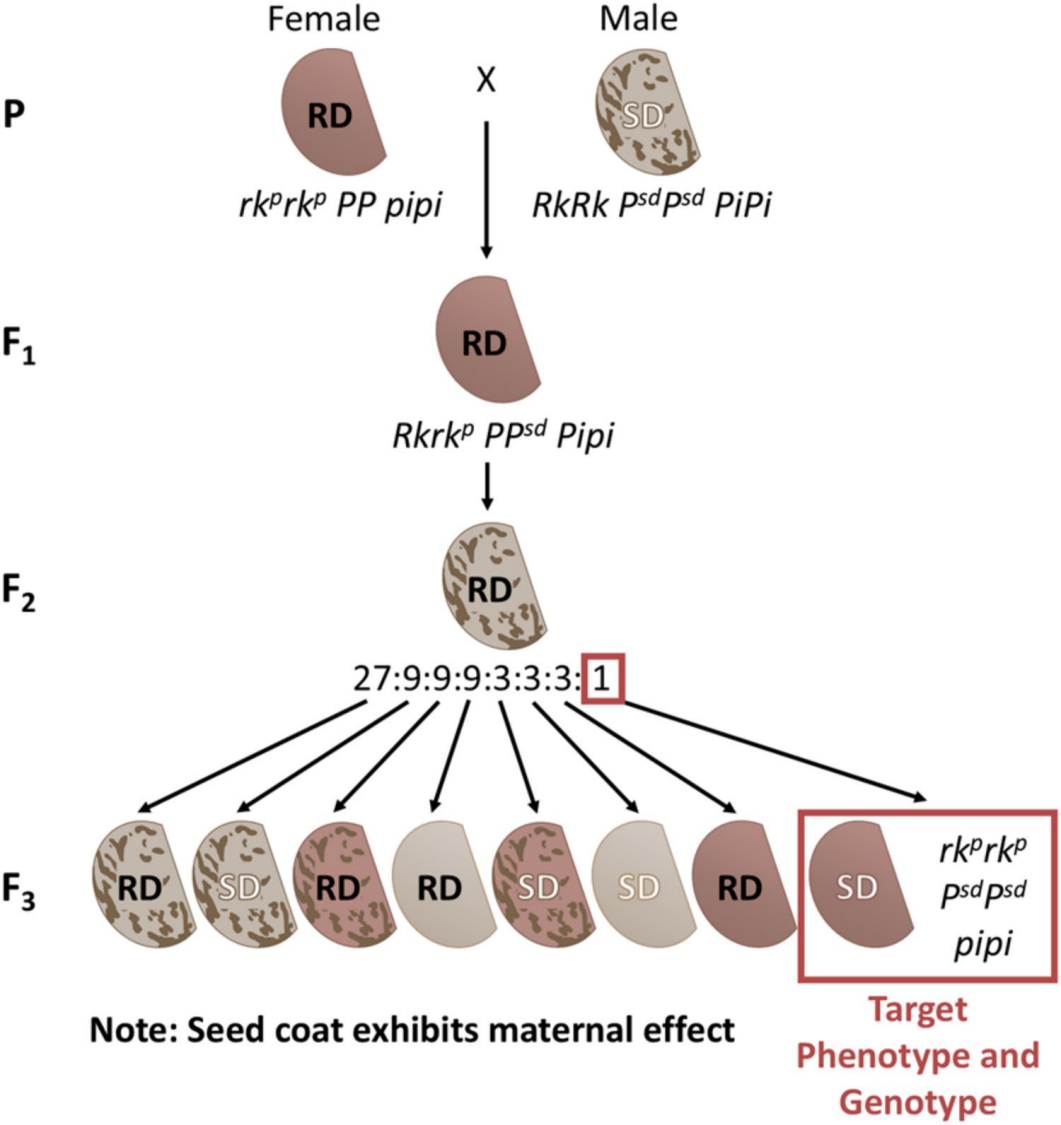
Crossing scheme to introduce the *P*^*sd*^ allele into pink beans. As seed coat exhibits maternal effect, the phenotype is dependent on the genotype of the mother. Genotype segregation begins in the F_2_ generation, while phenotypic segregation is not observed until the F_3_ generation. The scheme illustrates the introduction of three key alleles required to produce slow‑darkening (SD) pink beans: *P*^*sd*^ (slow‑darkening), *rk*^*p*^ (pink seed coat), and *pi* (non‑pinto pattern). RD = regular darkening

To introduce the slow-darkening (*P*^*sd*^*)* allele into pink bean backgrounds, crosses were made between the SD pinto cultivar ND Palomino and three pink bean genotypes: Rosetta, ND112929, and PK16-1 (Fig. 6). The F_2_ plants from these crosses were genotyped with the *Pi*, *rk*^*p*^, and *P*^*sd*^ markers to select individuals with the desired genotypic combination. The goal was to select F_2_ plants exhibiting slow-darkening pink bean phenotype, characterized by homozygous recessive alleles at all three loci: *P*^*sd*^, *rk*^*p*^, and *pi*. Of the 2240 F_2_ plants screened, 2,171 plants had successful genotyping results for all three markers. Among these, 32 individuals carried the target allelic combination predictive of slow-darkening pink phenotype. Total evaluated individuals from each cross are presented in Table 4, while genotype and their assumed phenotype associations are summarized in Supplementary Table S11. The segregation patterns for all three loci fit an expected Mendelian ratio, as supported by *χ*^*2*^ analysis (Supplementary Table S12).

**Table 4:** Crosses screened and the number of F₂ segregating SD pink progeny per cross

	Number of F_2_ plants screened	Number of SD pink plants	Number of viable SD pink lines^a^
Rosetta × ND Palomino	745	12	12
ND112929 × ND Palomino	780	11	10
PK16-1 × ND Palomino	715	9	6
Total	2240	32	28

^a^Four of the SD pink plants did not produce enough seed to move on to further stages of the breeding process

### UV testing

**Fig. 7:**
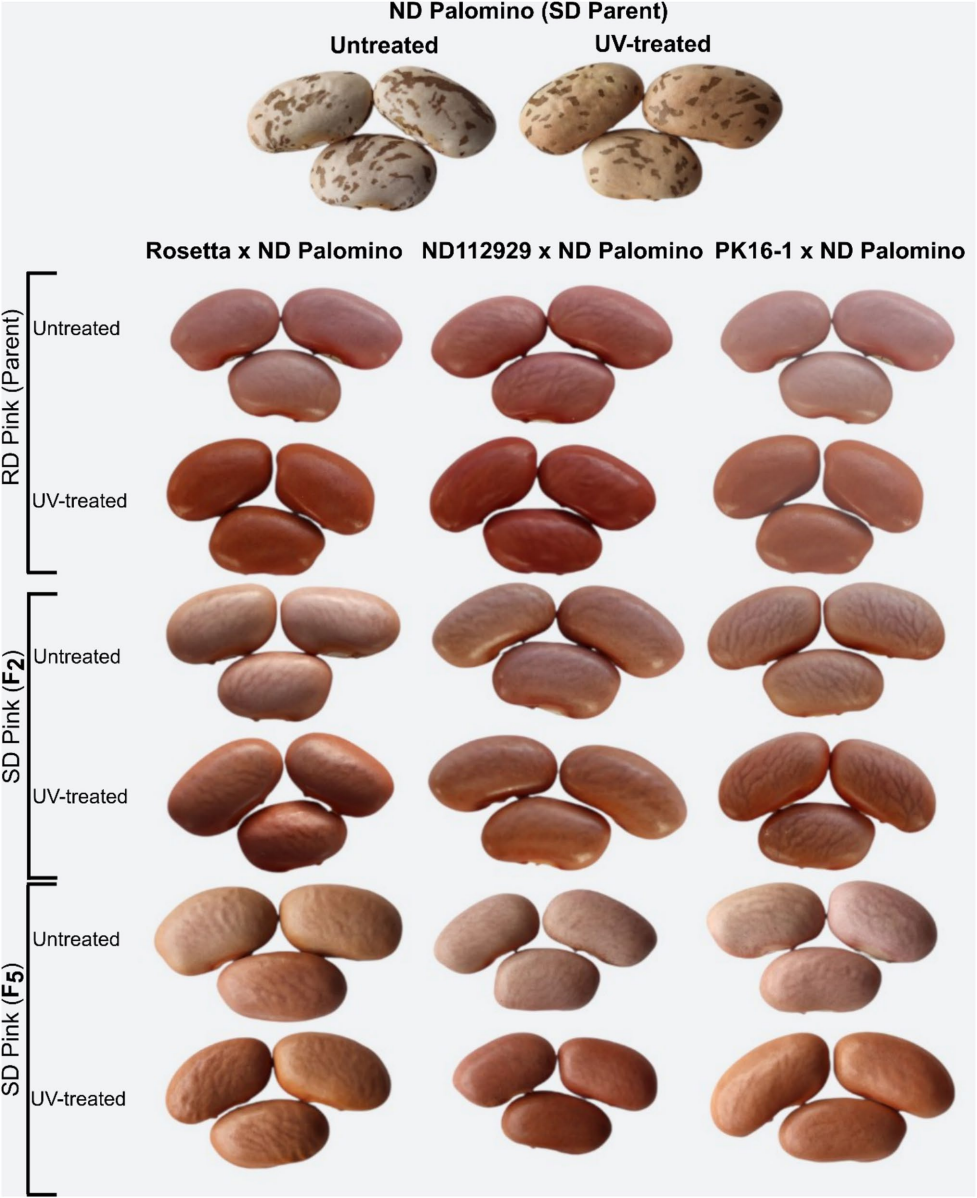
Ultra-violet (UV)-induced seed coat darkening test results in bean crosses**.** Representative seed samples from three crosses involving the slow-darkening (SD) parent ND Palomino are shown. The top panel displays untreated and UV-treated seeds of ND Palomino. Each subsequent panel corresponds to a cross with a regular-darkening (RD) pink parent (Rosetta, ND112929, and PK16-1), with untreated and UV-treated seeds from the RD parent (top row), F_2_ generation (middle row), and F_5_ generation (bottom row). UV treatment highlights variation in seed coat darkening across genotypes and generations

Seeds from the parental lines and F_2_ progeny were exposed to UV light for five days (120 h) to evaluate postharvest darkening. Following UV exposure, 28 slow-darkening pink lines were compared to both untreated seeds and their regular-darkening pink parent. This comparison revealed a clear phenotypic distinction in seed coat darkening between the SD and regular-darkening (RD) populations (Fig. 7). The selected F_2_ SD pink lines were advanced in the field to the F_5_ generation. Genotyping of these generations using PACE markers for *P*^*sd*^ and *rk*^*p*^ further validated the presence of the homozygous alleles associated with reduced darkening and pink seed coat color, respectively. UV-treated seeds from the F_2_ and F_5_ SD lines consistently exhibited reduced darkening, and visual inspection of F_5_-derived seeds confirmed retention of the characteristic pink seed coat color, indicating phenotypic stability across generations.

## Discussion

### Mapping and candidate gene selection for the *Rk* locus

The *Rk* gene is responsible for red kidney seed coat color in common bean and is known to regulate red pigmentation in LRKs, DRKs, pink, and Durango red beans (Bassett and Miklas 2003). The GWAS conducted in this study mapped the *Rk* locus within a narrow 0.8 Mb interval (39.3–40.1 Mb) near the end of chromosome Pv02. Candidate gene searches within this interval, focused on genes involved in flavonoid biosynthesis, revealed a pair of duplicated genes, PvUI111.02G213800 and PvUI111.02G213700, with identical annotation. The ortholog of these two genes in the model organism Arabidopsis is *AT1G61720*, known as the *BAN* gene. *BAN* is a negative regulator of flavonoid biosynthesis (Albert et al. 1997). *BAN* encodes anthocyanidin reductase (ANR), an enzyme in the flavonoid pathway that converts anthocyanidins into flavan-3-ols, which in turn are polymerized into condensed tannins (Xie et al. 2003). This enzymatic activity is important as it lessens the accumulation of red colored anthocyanidins, and the resulting brown tint from condensed tannins that further obscure red pigmentation (Liu et al. 2021).

The common bean *Rk* gene appears to function analogously to *BAN*, acting as a negative regulator of red pigment production. When a dominant *Rk* or *BAN* allele is present, there is no red color. This functional similarity strongly suggests that the two bean genes are orthologs of the Arabidopsis *BAN* gene. Although both bean genes share the same annotation, RNA-seq analysis of seed coat tissue revealed that only PvUI111.02G213800 is expressed during seed coat development. Therefore, PvUI111.02G213800 was selected as the most likely candidate for the *Rk* gene.

### Evidence supporting the candidate gene

Historically, members of the *Phaseolus* genus have shown resistance to the introduction of foreign DNA (Aragão and Rech 2001). Because creating transgenic common bean plants is challenging, alternative approaches must be used to verify gene function. In this study, multiple lines of evidence support PvUI111.02G213800 as the candidate gene for *Rk*, including genetic linkage mapping, phylogenetic analysis, segregation analysis in F_2_ populations, and analysis of missense SNP markers.

Genetic linkage mapping using GM RIL population confirmed the expected marker order based on physical positions in the reference genome. Most importantly, complete cosegregation between the *Rk* phenotype with a missense SNP, Pv02_39518891 PACE marker located within the coding region of PvUI111.02G213800, supports that this gene underlies the *Rk* phenotype.

Phylogenetic analysis of *ANR* orthologs across 57 species further supports PvUI111.02G213800 as the candidate gene underlying the *Rk* locus. The *Rk* protein exhibits extremely high sequence identity (98–99%) with orthologs from closely related *Phaseolus* species, reflecting strong conservation within the genus. Although identity values decline with increasing taxonomic distance, they remain high in other legumes such as cowpea (94%) and soybean (90%). The placement of *Rk* protein within a well-supported legume-specific *ANR* clade in the NJ tree reinforces its functional relevance in seed coat pigmentation pathways. Kovinich et al. (2012) demonstrated that soybean mutants with reduced expression of *ANR1* ortholog (Glyma.08G062000) exhibit a red seed coat phenotype. In cowpea, the gene model Vigun03g118700*,* a homolog of soybean *ANR1* and common bean *Rk,* was identified as a strong candidate controlling red seed coat color (Herniter et al. 2024). Together, these cross-species observations highlight the role of ANR-type enzymes in determining seed coat pigmentation across legumes. These findings, together with GWAS, gene expression, linkage mapping, and F_2_ segregation analyses, strengthen the support for PvUI111.02G213800 as the causal gene underlying the *Rk* locus.

### Alleles of *Rk*

Currently, four recessive alleles of the *Rk* gene are recognized: *rk*, *rk*^*p*^, *rk*^*d*^, and *rk*^*cd*^. In this study, sequence analysis of the candidate gene model PvUI111.02G213800 revealed that pink (*rk*^*p*^) and Durango red (*rk*^*cd*^) genotypes shared identical nucleotide and protein sequences. Both market classes carried the same missense SNP at 39,518,531 bp, resulting in an amino acid substitution that distinguishes them from the other seed coat types. This finding suggests that *rk*^*p*^ and *rk*^*cd*^ are not genetically distinct at the *Rk* locus. According to Bassett and Miklas (2003), the *rk*^*cd*^ manifests as a pink hue when paired with the genotype *c*^*u*^*c*^*u*^ and garnet brown (red hue) with *C*_. All pink beans carry *c*^*u*^*c*^*u*^, while Durango red beans carry *C*_, indicating the observed color differences may be due to variation at the *C* alleles rather than differences at *Rk*. Furthermore, *c*^*u*^*c*^*u*^ is known to protect the color of pink beans from the effects of *G B V* (Bassett and Miklas 2003), while Durango red beans are affected by *G B V* interactions. Durango red beans typically have the genotype *G b v*, indicating that *G* and *C* could contribute to the seed coat color variation between Durango red and pink beans rather than distinct *Rk* alleles. Based on this evidence, we propose retiring the *rk*^*cd*^ allele designation and using *rk*^*p*^ for both pink and Durango red beans. Moreover, the targeted GWAS comparing pink and Durango red beans identified a strong association peak on chromosome Pv08, corresponding precisely to the physical interval of the complex *C* locus. This result reinforces the Bassett and Miklas (2023) hypothesis that the *rk*^*p*^ allele interacts with the *C* gene to produce the characteristic dark red background of the Durango market class. In contrast, pink beans carry the *c*^*u*^*c*^*u*^ genotype, which suppresses the expression of this darker pigmentation even in the presence of *rk*^*p*^. These findings highlight the complementary roles of the *Rk* and *C*/*c*^*u*^ loci in determining seed coat background color. Importantly, the combined use of markers for the *rk*^*p*^ allele and the *C*/*c*^*u*^ locus provides a reliable molecular strategy for distinguishing pink beans from Durango red beans, enabling more precise selection in breeding programs.

The existence of the remaining recessive alleles, *rk* and *rk*^*d*^, has been supported by distinct missense SNPs in the *Rk* gene. Both LRK (*rk*) and DRK (*rk*^*d*^) genotypes share a missense SNP at 39,518,891 bp, are further distinguished by another missense SNP at 39,519,517 bp, which is unique to DRKs (*rk*^*d*^). The pigmentation differences between LRKs and DRKs are attributed to varying levels of proanthocyanidins. DRKs exhibit a garnet brown color due to the higher accumulation of proanthocyanidins (Beninger and Hosfield 1999), while LRKs display a testaceous color as a result of reduced concentration of proanthocyanidins (Bassett 2007). These differences are consistent with the hypothesis that the *rk*^*d*^ allele results in moderately reduced ANR expression, allowing sufficient red pigment accumulation for the garnet brown color found in DRKs. In contrast, the *rk* allele likely causes further suppression of ANR expression compared to *rk*^*d*^, resulting in lighter pigmentation to get the testaceous color found in LRKs as well as preventing the color from darkening to garnet brown from the production of a higher amount of proanthocyanidins. The *rk*^*p*^ allele, associated with pink beans, is likely to cause even lower ANR expression, producing the pale pink color phenotype. Durango red beans, although carrying *rk*^*p*^, have a darker seed coat color due to modifying effects of the *C* loci.

Based on these findings, we propose a revised designation for the *Rk* alleles: *Rk*, *rk*, *rk*^*d*^, and *rk*^*p*^, with *rk*^*cd*^ subsumed under *rk*^*p*^. Each recessive allele is defined by a unique missense SNP and is traceable using corresponding functioning PACE marker. The revised order of dominance suggested by Bassett and Miklas (2003) (accounting for the removal of *rk*^*cd*^) is *Rk* > *rk* > *rk*^*p*^ > *rk*^*d*^.

### Development and validation of diagnostic PACE markers for *Rk* allele differentiation

All three diagnostic SNPs were successfully converted into market class specific PACE markers. The *rk*^*p*^ (39,518,531 bp) PACE marker, specific to pink and Durango red bean seed type, was fully diagnostic across MDP and ADP panels, clearly differentiating these classes from others. The shared LRK and DRK marker (39,518,891 bp) was absent in MDP genotypes and present in both LRK and DRK genotypes in the ADP panel, with few exceptions. These exceptions may be attributable to seed type misclassification or residual heterozygosity, as bean breeders often cross between gene pools, races, and multiple market classes within the same gene pools. The LRK and DRK genotypes were further separated by the DRK-specific PACE marker (39,519,517 bp) which reliably identified DRK genotypes in the ADP panel.

In contrast, the markers developed for red mottled and small red beans (syn. Central American reds) did not properly identify their respective market classes. The red coloration of red mottled beans is likely controlled by the *R* gene within the complex C locus [*C*^*st*^* R*] rather than *Rk*, explaining the lack of diagnostic power. Similarly, the small red markers correspond to silent mutations that did not affect the phenotype and therefore were not consistently present across all small red beans.

### Introduction of the ***P***^***sd***^ allele successfully into pink beans

In this study, we successfully introduced the *P*^*sd*^ allele into pink bean backgrounds through intermarket class crosses and marker-assisted selection. The donor parent, ND Palomino, a slow-darkening pinto variety, was crossed with three pink bean genotypes. ND Palomino was used as the male parent in all the crosses to ensure that seed coat phenotype of the F_1_ generation reflected the maternal genotype, as the seed coat is derived from maternal tissue (Singh et al. 2017), so seed coats have the genotype and phenotype of the mother. As expected, the F₁ seeds displayed the pink and unmottled phenotype of the female parent, while the F₂ generation segregated for seed coat traits, including the desired SD trait.

To efficiently identify SD pink individuals, we employed PACE markers targeting *P*^*sd*^, *rk*^*p*^, and *pi.* This genotypic selection strategy enabled early identification of F_2_ plants homozygous for three recessive alleles associated with the SD pink phenotype, effectively excluding the pinto-patterned phenotypes. A total of 32 F_2_ individuals (28 viable) were selected based on the desired three PACE marker profiles, all of which exhibited SD pink phenotype, confirming the diagnostic accuracy of the marker sets. This strategy circumvented the need to wait for seed production to assess phenotype, highlighting the utility of marker-assisted selection in overcoming maternal masking effects and accelerate breeding progress. The selected F_2_ plants were advanced to the F_5_ generation through field trials. UV testing and visual inspection of F_2_^-^ to F_5_-derived seeds confirmed the retention of the characteristic pink seed coat color, indicating that the SD phenotype remained stable across generations. Genotyping these generations further validated the presence of homozygous *P*^*sd*^, *rk*^*p*^, and *pi* alleles, reinforcing the association between genotype and phenotype.

These results demonstrate the feasibility and effectiveness of introducing the *P*^*sd*^ allele into pink beans using marker-assisted selection. The approach can serve as a model for transferring the SD trait into other susceptible LRK and cranberry market classes, ultimately contributing to improved seed quality and consumer acceptance across the dry bean crop.

## Conclusions

This study accomplished two major goals: identifying a strong candidate gene for *Rk* and applying that discovery to introduce the slow-darkening (*P*^*sd*^) trait into pink beans. Given the vulnerability of pink beans to postharvest seed coat darkening, the development of SD pink germplasm represents a significant advancement toward improving seed quality and market competitiveness. Through an integrated approach combining GWAS, gene expression profiling, phylogenetic analysis, and genetic linkage mapping, PvUI111.02G213800, encoding anthocyanidin reductase, was selected as the most compelling candidate gene for *Rk*. This gene model was further supported by its functional annotation, expression in seed coat tissue, and the cosegregation of the phenotype with a marker located within the gene. A suite of diagnostic PACE markers linked to specific *Rk* alleles were developed from missense SNPs within the *Rk* candidate gene, demonstrating their diagnostic accuracy across diverse germplasm panels (ADP and MDP). The *rk*^*p*^ allele marker was particularly instrumental for the successful introduction of the SD trait into pink beans.

The future implications of this research look promising. The newly developed SD pink lines, although not agronomically suitable for commercial release, will serve as a germplasm resource for future breeding efforts to develop SD pink cultivars. Additionally, preliminary work has begun to extend the SD trait into LRK and cranberry beans using marker-assisted selection for seed color and patterning genes, broadening the potential impact of this trait across multiple dry bean market classes.

## Supplementary Information

Below is the link to the electronic supplementary material.

Supplementary file1 (DOCX 2296 KB)

Supplementary file2 (XLSX 89 KB)

## Data Availability

The datasets generated and/or analyzed during the current study are available from the corresponding author on reasonable request. Some of the data are shown in Supporting Information.
