# Supplementary material for: A candidate gene marker at the red kidney color locus (Rk) enables the development of slow-darkening pink beans
Source: Theor Appl Genet. 2026 May 20;139(6):157. doi: 10.1007/s00122-026-05256-z (PMC13190465; doi:10.1007/s00122-026-05256-z)
Supplement: Supplementary file 1 — Supplementary file1 (DOCX 2296 KB) [file 122_2026_5256_MOESM1_ESM.docx]

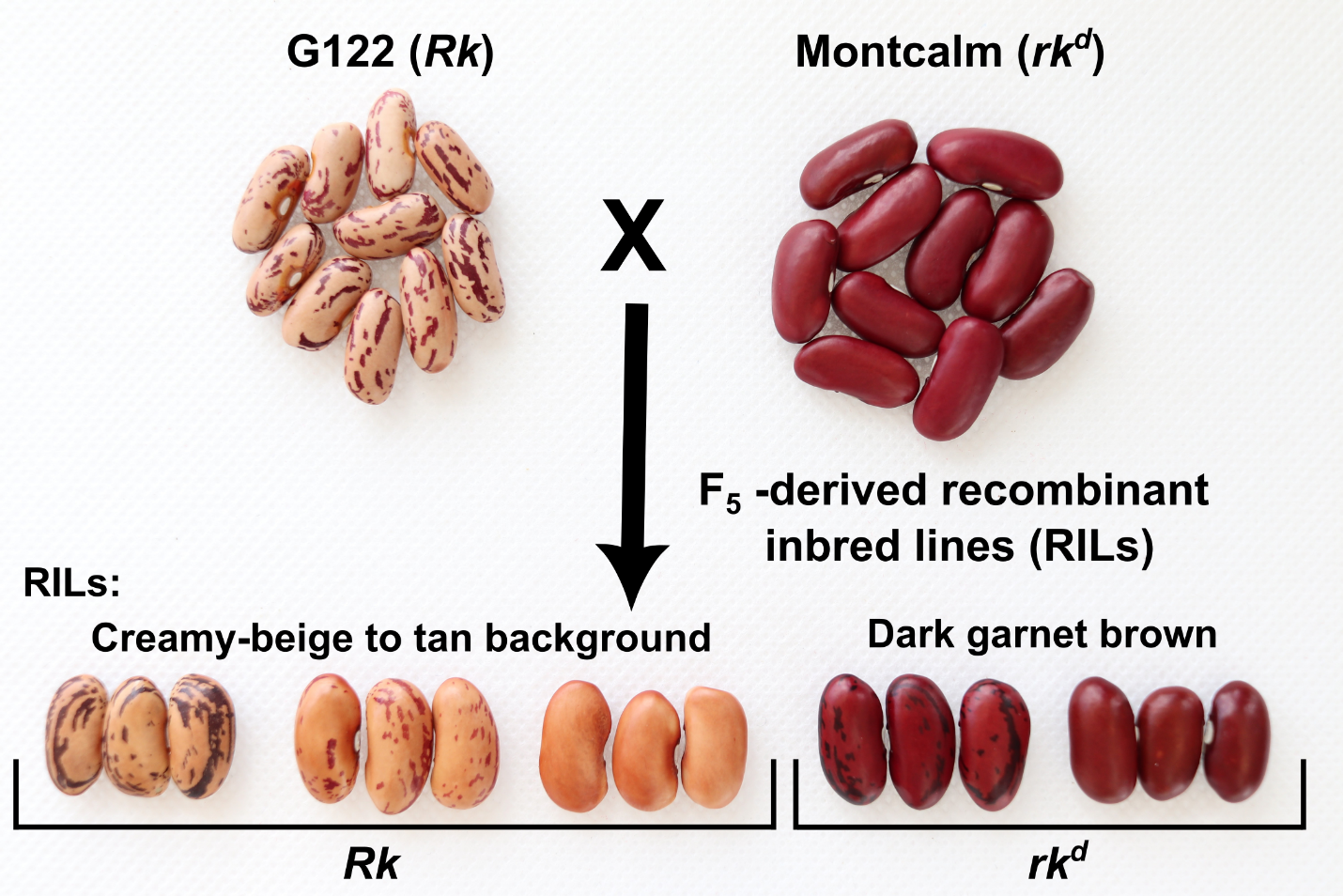


**Supplementary Fig. S1** Segregation of seed coat phenotypes in F₅‑derived recombinant inbred lines (RILs) from the cross G122 × Montcalm. G122 (*Rk*) exhibits a light creamy-beige background with red striped and mottled seed coat patterns, whereas Montcalm (*rk^d^*) displays a uniform dark garnet‑brown seed coat. The F₅‑derived RIL population segregated into two distinct phenotypic classes: *Rk* lines showing solid light creamy-beige to tan background seed coat, sometimes with striped and mottled patterns; and *rk^d^* lines exhibiting either solid dark garnet-brown or dark garnet-brown seeds with striped and mottled patterns.


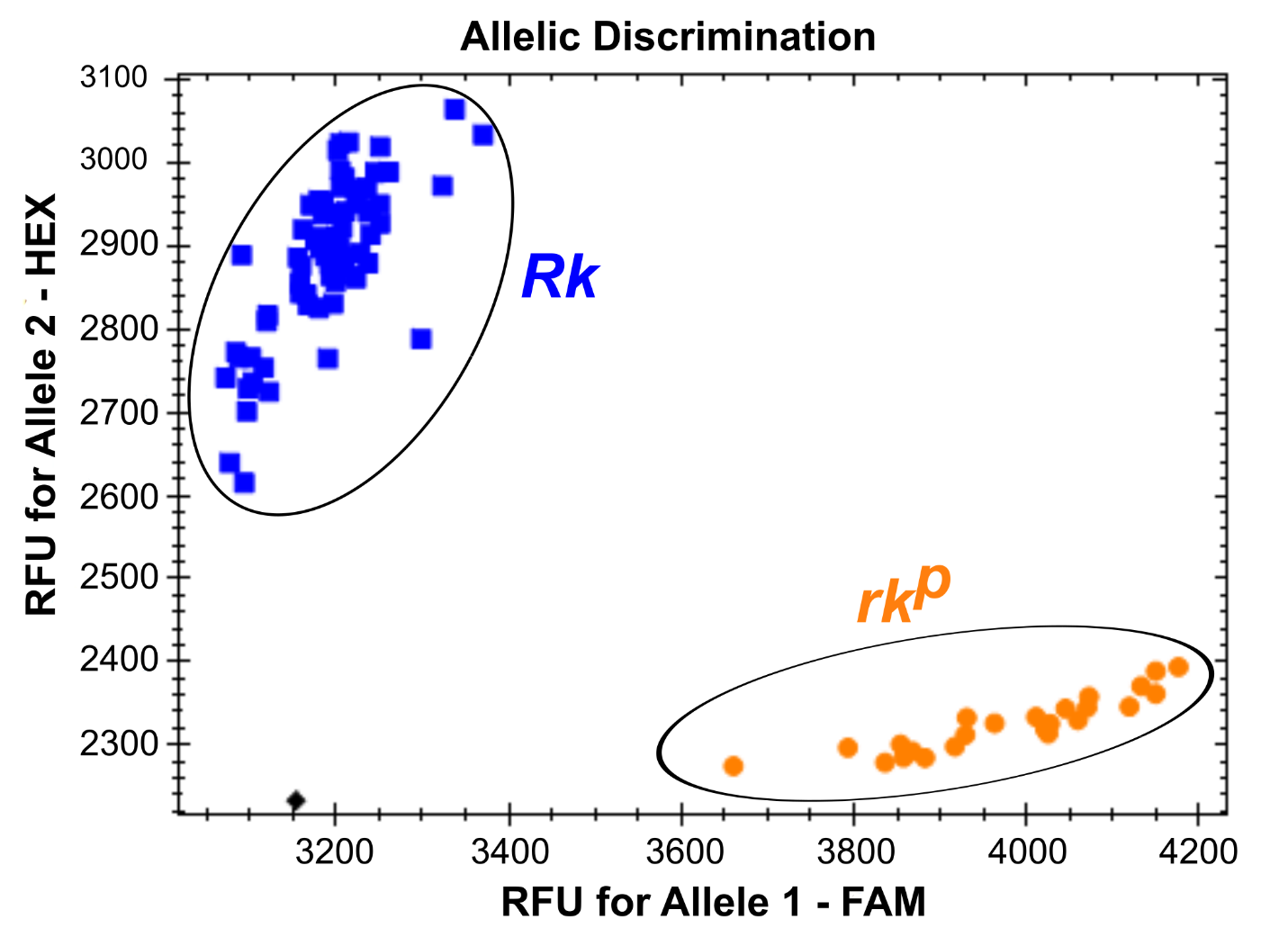


**Supplementary Fig. S2** Allelic discrimination plot for the *rk^p^* PACE marker (*rk^p^*-PACE-PinkFloyd-SNP-39518531-UI111). Fluorescence intensities (RFU) for the FAM‑ and HEX‑labeled probes clearly separate the two allelic classes. Samples carrying the *Rk* allele (Allele 2) cluster distinctly from those carrying the *rk^p^* allele (Allele 1), demonstrating robust genotypic discrimination and reliable marker performance. Data shown are from a 96‑well plate of the MDP panel, consisting of 95 samples and one no‑template control (NTC), represented by the black diamond.


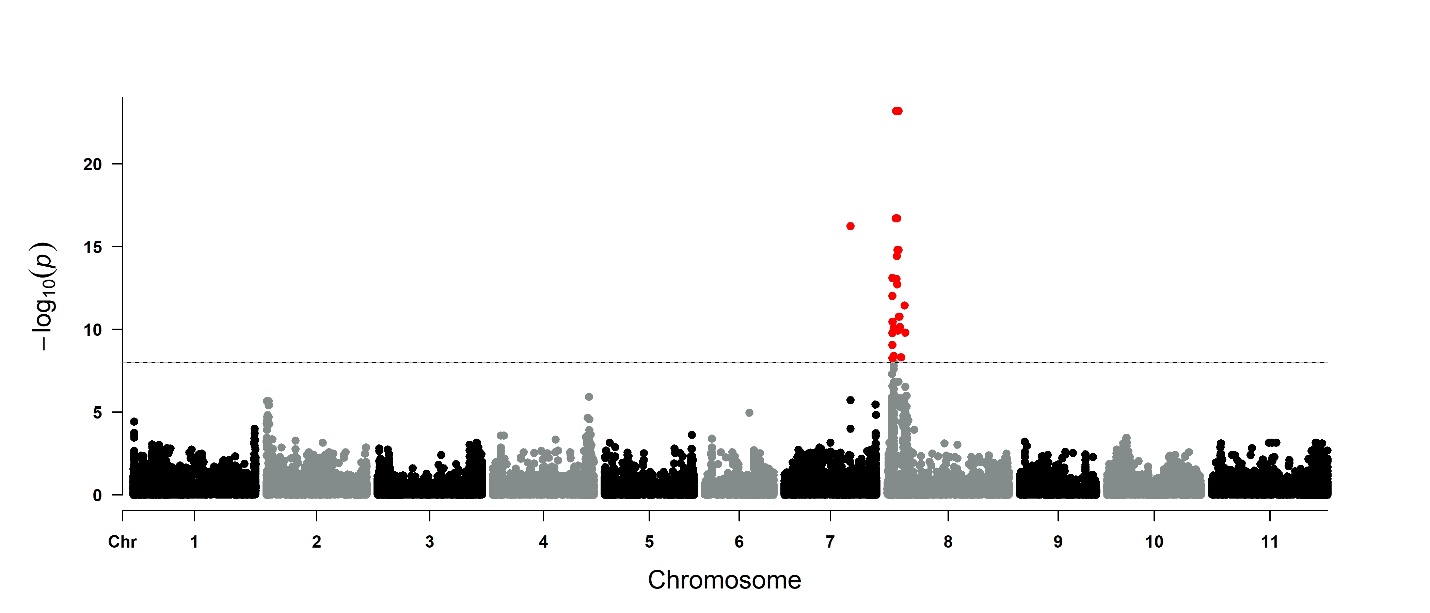


**Supplementary Fig. S3** Manhattan plot showing genomic regions associated with seed coat background color based on a GWAS comparing solid‑colored pink and Durango red bean genotypes. A prominent association peak is observed on chromosome 8, corresponding to the physical interval of the complex *C* locus, and an additional peak is detected on chromosome 7. The dotted horizontal line indicates the genome‑wide significance threshold.
